# Gender, Age, and Education Level Modify the Association between Body Mass Index and Physical Activity: A Cross-Sectional Study in Hangzhou, China

**DOI:** 10.1371/journal.pone.0125534

**Published:** 2015-05-05

**Authors:** Mengyu Fan, Meng Su, Yayun Tan, Qingmin Liu, Yanjun Ren, Liming Li, Jun Lv

**Affiliations:** 1 Department of Epidemiology and Biostatistics, School of Public Health, Peking University Health Science Center, Beijing, 100191, China; 2 National Clinical Research Center of Cardiovascular Diseases, State Key Laboratory of Cardiovascular Disease, Fuwai Hospital, National Center for Cardiovascular Diseases, Chinese Academy of Medical Sciences and Peking Union Medical College, Beijing, China; 3 Department of Chronic Non-communicable Disease Control and Prevention, Hangzhou Center for Disease Control and Prevention, Hangzhou, 310021, China; MOE Key Laboratory of Environment and Health, School of Public Health, Tongji Medical College, Huazhong University of Science and Technology, CHINA

## Abstract

**Introduction:**

Numerous studies have reported a strong inverse association between BMI and physical activity in western populations. Recently, the association between BMI and physical activity has been considered bidirectional. This study aimed to examine the associations of body mass index (BMI) with physical activity and sedentary behavior and to explore whether those associations were modified by socio-demographic characteristics.

**Materials and Methods:**

We conducted a multistage random sampling survey in three districts of Hangzhou, China, in 2012. The International Physical Activity Questionnaire long form was used to collect data regarding physical activity and sedentary behavior. A multilevel mixed-effects regression model was used to assess the associations of BMI with physical activity and sedentary behavior.

**Results:**

A total of 1362 eligible people (624 men and 738 women, ages 23-59 years) completed the survey. People who are young or middle-aged and have the highest education level are the most inactive. Significant differences in the associations between physical activity and BMI across socio-demographic groups were identified (sex*BMI, P=0.018; age*BMI, *P*<0.001; education level*BMI, *P*=0.030). Women or individuals older than 50 had a higher level of physical activity with increasing BMI. There was no statistically significant association between BMI and sedentary behavior (*P*=0.450).

**Conclusions:**

The associations between BMI and physical activity were modified by sex, age, and education level in Hangzhou, China.

## Introduction

The global rise of overweight and obesity has serious health effects, such as cardiovascular diseases, type 2 diabetes, osteoarthritis, some cancers, and a higher risk of all-cause mortality [1]. Increasing physical inactivity and prolonged sedentary time, together with an excess energy intake, contribute to the emerging overweight and obesity epidemic [1,2]. Discussions regarding the associations of obesity with physical activity (PA) and sedentary behavior (SB) are not new. Physical inactivity and sedentary behavior have long been considered to lead to overweight and obesity [3,4,5]. However, a diametrically opposite view—that the associations between PA/SB and overweight and obesity was bidirectional or reverse causality—have prevailed in recent years. Some studies have shown no evidence that physical inactivity promotes the development of obesity but indicated that overweight and obese individuals tend to be physically inactive [6,7,8,9]. Heavier individuals may be more sedentary than lighter-weight individuals partly because physical activity is less pleasurable for heavier people and partly because of the embarrassment that heavier people feel regarding being seen in public in exercise clothes. Some studies even showed a potential gender difference [6,7,10]. Alternatively, high body mass index (BMI) can also be a motivator for initiating exercise. The common explanations for these positive associations are body dissatisfaction and health consciousness. One of the most frequent reasons that individuals give for physical activity is losing weight, which is largely motivated by an increase in body weight or a decline in health status [11,12]. Body dissatisfaction and health consciousness may increase as the degree of obesity increases [11,13], which would encourage heavier individuals to participate in more physical activities. In addition, the degree of two potential motivators is related to socio-demographic characteristics, such as female gender, younger age, increased level of education, and level of overweight [11,12,14,15].

However, the above evidence was primarily derived from the studies conducted in Western populations, many of which assessed leisure-time physical activity only; however, physical activity obtained in other domains may be crucial for China due to the rather low (<20%) proportion of leisure-time physical activity participation among Chinese adults [16,17]. It should be also noted that there are great societal and cultural differences between China and Western countries. This article has two objectives using a representative sample of Chinese urban adults aged 20–59 years in Hangzhou City, China: 1) to investigate the associations between BMI and physical activity/sedentary behavior in Chinese urban population and 2) to explore whether these associations are modified by socio-demographic characteristics.

## Materials and Methods

### Participants and study design

This study was conducted in Hangzhou, the capital of Zhejiang province, from June to December 2012. Three districts in Hangzhou were included in this study: Shangcheng, Xiacheng, and Xihu.

The eligible subjects were individuals aged 20–59 who had lived in the district for at least one year. Individuals reporting any sickness or disability that affected their normal activity over the last 7 days were excluded. A multistage random sampling strategy with stratification by functional unit was used in this study. The definition of the functional unit has been described elsewhere [18]. Briefly, there are five categories in these three districts based on the degree of land-use mix and public services capacity, and three units were included in this study. Thirty of the 170 communities (ten communities in each functional unit) were randomly selected. In each selected community, a random sample of households was taken from lists of community households. In each selected household, one of the eligible persons was identified using the Kish method. All interviewers were asked to have a maximum of three door-to-door visiting attempts per sampled household. A total of 2570 households were selected for participation, and 1440 people completed the survey. Overall, 1362 participants were eligible for analysis (78 subjects were excluded because of incomplete data).

Face-to-face interviews were conducted by trained local CDC staff. A two-day training procedure, which included explanation of the questionnaire and a pre-investigation of 3 to 5 subjects, was given before the beginning of the investigation. The study was approved by the Peking University Institutional Review Board (Certificate Number: IRB00001052-11030). Written informed consent ensuring privacy and confidentiality was obtained from participants.

### Measures and variables

The questionnaire consisted of 4 major sections related to general demographic and socioeconomic status, physical activity, sedentary behavior and anthropometric variables.

Basic socio-demographic information was collected from all participants: sex, age, education, work status, and income. We grouped age into four categories: <30 years, 30–39 years, 40–49 years, and ≥50 years. We grouped education into eight categories: 1) no formal schooling, 2) primary school completed, 3) junior high school completed, 4) senior high school completed, 5) sedentary vocational school, 6) three-year college, 7) college/university completed, and 8) post-graduate degree completed. Work status was grouped into “employed” or “unemployed”. We calculated per capita monthly income based on the household size and total annual income of the household. Based on the Basic Statistics on Urban Households (2012), we grouped the participants into three categories: low (RMB 0–999), medium (RMB 1,000–3,999), and high (RMB ≥ 4,000) levels.

The questions on physical activity and sedentary behavior were modeled after the International Physical Activity Questionnaire long format (IPAQ-L) [19]. Data cleaning and processing of physical activity and sedentary behavior strictly adhered to the formal guidelines proposed by the IPAQ core group [20]. Physical activity was measured in four domains: work, active transportation, domestic and garden, and leisure time. For each domain of activity, participants were asked about the number of days and hours/minutes per day over the last week related to walking, moderate-intensity, and vigorous-intensity activity. Metabolic equivalent tasks (METs) from the formal guidelines of IPAQ-L were used. The number of minutes spent per week participating in each activity was multiplied by the MET score for that activity, and the weekly amount of domain specific physical activity was obtained by summing the MET-minutes for activities related to work, transport, housework, and leisure time activities. An overall total physical activity MET-minutes/week score can be computed using the following equation: Total physical activity MET-minutes/week = sum of Total (Work + Transport + Housework + Leisure-Time) MET-minutes/week scores. Sedentary time, which is not included as part of any physical activity calculation, was quantified as minutes/week. We calculated total sedentary time/week based on an estimate of sitting on a typical weekday, weekend day, and time spent sitting during travel.

Body weight and standing height were collected by self-report. BMI was calculated as weight (in kg) divided by the square of standing height (in m) and categorized according to standard cut-points for Asian populations (underweight ≤ 18.5, normal weight = 18.5–< 24.0, overweight = 24.0–< 28.0, and obese = ≥ 28.0) [21].

### Statistical analysis

Descriptive analyses were based on frequencies and percentages for categorical variables. For continuous variables, we used median values with the interquartile ranges (IQRs) for description due to their non-normal distributions. Chi-square tests were used to compare two or more groups between genders. Nonparametric rank sum tests were used to differentiate the physical activity scores/ sedentary time from socio-demographic variables. Comparisons of the variables between two independent samples were performed using the Wilcoxon rank-sum test, and the Kruskal-Wallis rank test was used for comparisons among more than two groups.

Multilevel mixed-effects regression models with a random effect at the community level were performed to assess the associations between physical activity and sedentary behavior and BMI, considering the individual’s variables to be Level 1 and the community variable to be Level 2. We selected the continuous values of total physical activity (TPA), leisure-time physical activity (LTPA), and sedentary time (ST) as outcome variables. Among them, LTPA is considered to evaluate an individual’s voluntary physical activity. Given the non-normal distribution of the residuals in the multilevel linear regression models and 53 participants stated no physical activity at all (physical activity score = 0), before running the regression analysis, the TPA and LTPA scores were both log transformed after adding 100 (LN (PA+100)). Models were adjusted for sex, age, education level, occupation and interviewed month of the participants. The models for LTPA were further adjusted for work, transportation, and housework physical activity. To explore the modification of socio-demographic characteristics, the interactions between gender and BMI, age and BMI, and education level and BMI were included in multilevel regression models. We used likelihood ratio tests to compare multilevel linear regression models with and without the interaction term for BMI (both treated as continuous variables) and the socio-demographic factor. The income variable was not included in the analysis because nearly one third of the data were missing. All analyses were performed in 2014 using STATA 12.0 software and are two sided, with a significance level of *p*<0.05.

## Results

Table 1 presents descriptive statistics and statistical analyses of the socio-demographic characteristics of 1,362 participants between genders. Among all participants, 624 (45.8%) were men, with a mean BMI of 23.6±2.9 kg/m^2^, and 738 (54.2%) were women, with a mean BMI of 21.9±2.8 kg/m^2^. The median age was 43.0 years, and 75.2% of the entire sample was employed. Men had a higher level of educational attainment and a lower proportion of unemployment than women. There was no statistically significant difference in income level between men and women.

**Table 1:** Main characteristics of 1,362 participants.

Characteristics		Men		Women		*P*
		N	%	N	%	
Age	<30 y	70	11.2	92	12.5	0.021
(N = 1362)	30–39 y	180	28.8	191	25.9	
	40–49 y	199	31.9	197	26.7	
	≥50 y	175	28	258	35	
Education (N = 1360)	Junior high school or below	134	21.5	233	31.6	<0.001
	Senior high school	155	24.9	166	22.5	
	College/university or above	334	53.6	338	45.9	
Income	Low	47	10.3	50	9.9	0.829
(N = 960)	Medium	295	64.6	334	66.4	
	High	115	25.2	119	23.7	
Work status	Employed	558	89.5	466	63.1	<0.001
(N = 1361)	Unemployed	65	10.5	272	36.9	
BMI (kg/m^2^)		23.6±2.9		21.9±2.8		<0.001
(N = 1360)	<18.5	15	2.4	68	9.2	<0.001
	18.5–23.9	349	55.9	511	69.4	
	24.0–27.9	213	34.1	132	17.9	
	≥28.0	47	7.5	25	3.4	

Table 2 shows the level of total and four domains of physical activity and sedentary time by socio-demographic characteristics of all study participants. The median level of TPA was 2769 (IQRs: 3759) MET-minutes/week, and there was no gender difference. Differences in TPA were found across the other groups. Individuals older than 50 who had less education and lower income levels and were unemployed were more active. This trend was observed for both normal weight and overweight individuals. The median level of LTPA was 528 (IQRs: 1293) MET-minutes/week. Unemployed, normal weight and overweight individuals reported higher LTPA levels. The median sedentary time was 2010 (IQRs: 1393) minutes/week, and men tended to spend more time sitting than did women. For both men and women, sedentary time was associated with age (men, *p* = 0.002; women, *p*<0.001), attained educated level (men, *p*<0.001; women, *p*<0.001), and income (men, *p*<0.001; women, *p* = 0.023). Particularly for women, sedentary behavior was different across BMI levels (*p* = 0.033), indicating that underweight individuals were less active.

**Table 2:** Self-reported TPA and physical activity in each domain and sedentary time by socio-demographic variables*.

		TPA (MET-minutes/week)	Domain Sub Scores (MET- minutes/week)				Sedentary Time (minutes /week)
			Work	Transportation	Housework	Leisure-time	
Total		2769 (3759)	0 (1050)	765 (1414)	360 (1190)	528 (1293)	2010 (1393)
Sex	Men	2603 (3978)	330 (1980)	630 (1163)	180 (630)	480 (1188)	2118 (1328)
(N = 1362)	Women	2876 (3429)	0 (495)	900 (1424)	600 (1080)	594 (1304)	1850 (1320)
	*P*	0.585	<0.001	<0.001	<0.001	0.142	<0.001
Age	<30	1932 (3623)	281 (1808)	657 (1103)	90 (450)	396 (1083)	2258 (1279)
(N = 1362)	30–39	2511 (3534)	297 (1485)	600 (1059)	300 (900)	579 (1101)	2250 (1320)
	40–49	2658 (3854)	215 (1395)	720 (1449)	300 (720)	495 (1203)	2070 (1258)
	≥50	3324 (3596)	0 (462)	1260 (1733)	630 (1305)	693 (1386)	1680 (1245)
	*P*	<0.001	<0.001	<0.001	<0.001	0.336	<0.001
Education	Low	3450 (3699)	0 (693)	1260 (1800)	630 (1350)	693 (1386)	1680 (1200)
(N = 1360)	Medium	2979 (4088)	0 (1428)	840 (1461)	360 (1080)	396 (935)	1800 (1260)
	High	2332 (3373)	264 (1197)	600 (1140)	240 (720)	495 (1190)	2310 (1208)
	*P*	<0.001	<0.001	<0.001	<0.001	0.381	<0.001
Income	Low	3840 (4914)	0 (1587)	1260 (1836)	720 (2040)	693 (1566)	1610 (1280)
(N = 960)	Medium	2846 (3928)	66 (1386)	855 (1497)	360 (1080)	594 (1209)	1980 (1395)
	High	2070 (3113)	132 (738)	558 (1020)	233 (630)	398 (1143)	2280 (1231)
	*P*	<0.001	0.626	0.001	0.001	0.189	<0.001
Work status	Employed	2546 (3646)	396 (1869)	600 (1140)	265 (720)	438 (1031)	2130 (1320)
(N = 1361)	Unemployed	3450 (3546)	—	1302 (1872)	1035 (1943)	693 (1434)	1680 (1245)
	*P*	<0.001		<0.001	<0.001	<0.001	<0.001
BMI	<18.5	1983 (2997)	0 (990)	765 (1080)	225 (540)	330 (876)	2220 (1650)
(N = 1360)	18.5–23.9	2880 (3740)	33 (1095)	720 (1446)	420 (1260)	594 (1287)	2040 (1380)
	24.0–27.9	2760 (3735)	0 (1386)	855 (1451)	240 (1080)	594 (1386)	1960 (1350)
	≥28.0	2391 (4876)	0 (1287)	747 (1481)	265 (825)	371 (1133)	1850 (1439)
	*P*	0.002	0.85	0.493	<0.001	0.025	0.166

* Results are presented as median (IQRs).

TPA, total physical activity; BMI, body mass index.

**Fig 1:**
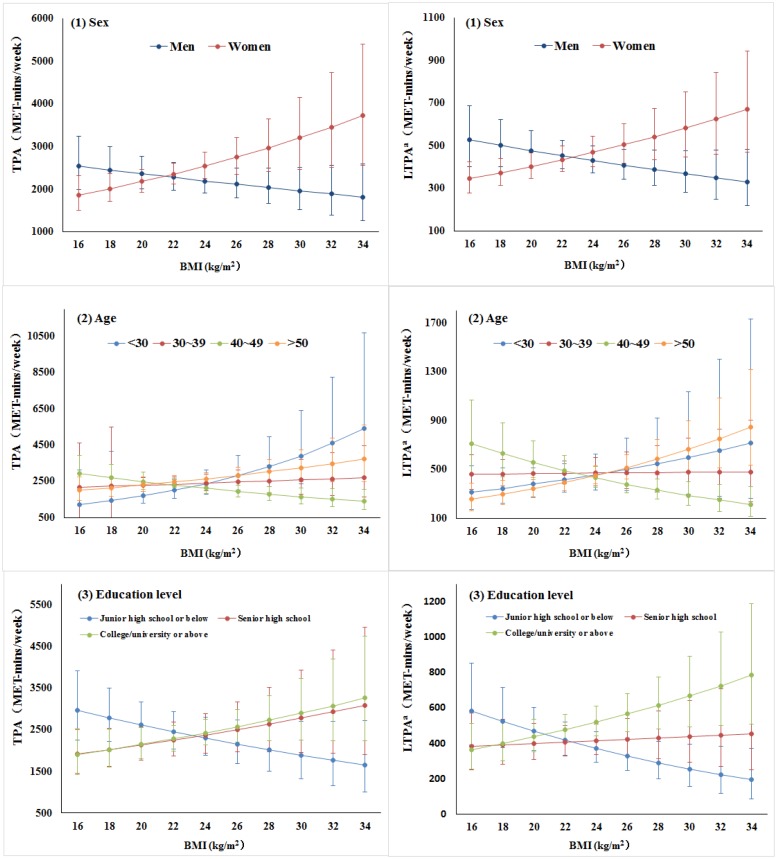
Associations between BMI and TPA/LTPA across sex, age, and education level groups; after grouping by community . TPA and LTPA in this figure were calculated as e^M^-100 and e^N^-100 separately, where M and N is the adjusted mean calculated using a multilevel mixed-effects model.

Table 3 displays the results of the multilevel regression analyzing the associations between BMI and TPA/LTPA. There was no statistical evidence of BMI and TPA in the total group. However, we observed statistically significant interactions between BMI and sex (P_interaction_ = 0.018), between BMI and age (P_interaction_<0.001) and between BMI and education level (P_interaction_ = 0.030). Among women, a positive association was observed between BMI and TPA. However, BMI had a weak, non-statistically significant negative association with TPA among men. Across the four age groups, strong negative association was found in the middle-aged groups (40–49 years). When considering the education groups, there was not a clear statistically significant association between BMI and TPA, although heavier adults with lowest level of education tended to be less active, whereas the other two education level groups tended to exercise more with increasing BMI (Fig 1).

**Table 3:** Multilevel regressions between BMI and TPA/LTPA stratified by socio-demographic category.

	TPA			LTPA					
				^†^ Model 1			^††^ Model 2		
	Coef.	95%CI	P	Coef.	SE	P	Coef.	SE	P
**Total participants**	0.011	-0.008,0.032	0.252	0.005	-0.017,0.028	0.642	0.002	-0.020,0.024	0.846
**Sex**									
Men	-0.015	-0.045,0.014	0.311	-0.018	-0.046,0.010	0.202			
Women	0.027	0.001,0.053	**0.043**	0.021	-0.001,0.042	0.059			
	**P**_**interaction**_ **= 0.018** ^§^			**P**_**interaction**_ **= 0.046** ^§^			P_interaction_ = 0.090^§^		
**Age**									
<30	0.057	-0.021,0.135	0.152	0.027	-0.043,0.096	0.449	0.010	-0.049,0.070	0.737
30–39	0.013	-0.019,0.046	0.423	0.022	-0.018,0.062	0.275	0.020	-0.015,0.055	0.258
40–49	-0.052	-0.083,-0.021	**0.001**	-0.072	-0.111,-0.033	**<0.001**	-0.073	-0.112,-0.033	**<0.001**
≥50	0.019	-0.013,0.052	0.248	0.037	0.001,0.074	**0.045**	0.035	-0.0004,0.070	**0.048**
	**P**_**interaction**_ **<0.001** ^‡^			**P**_**interaction**_ **= 0.001** ^‡^			**P**_**interaction**_ **= 0.003** ^‡^		
**Education**									
Junior high school or below	-0.026	-0.056,-0.003	0.079						
Senior high school	0.005	-0.029,0.039	0.761						
College/university or above	0.028	-0.005,0.061	0.100						
	**P**_**interaction**_ **= 0.030** ^||^			P_interaction_ = 0.051^||^			P_interaction_ = 0.0883^||^		

TPA, total physical activity; LTPA, leisure-time physical activity; BMI, body mass index.

^†^ Model 1: Grouping by Community; adjusted for sex, age, education level, occupation and interviewed month of the participants

^††^ Model 2: additionally adjusted for levels of work, transportation and housework physical activity

^§^ Test for interaction using the likelihood ratio tests to compare multilevel linear regression models with and without the interaction term for BMI (continuous) and sex (0, 1). The interaction terms were included in multilevel regression models that also controlled for age, education level and other factors mentioned above.

^‡^ Test for interaction using the likelihood ratio tests to compare multilevel linear regression models with and without the interaction term for BMI (continuous) and age (1, 2, 3, 4).

^||^ Test for interaction using the likelihood ratio tests to compare multilevel linear regression models with and without the interaction term for BMI (continuous) and education (1, 2, 3).

Furthermore, of the socio-demographic variables, sex and age had significant roles in modifying the associations between BMI and LTPA. A strong negative associations was observed in the 40–49 years age group. For the subgroups over the age of 50, the observed tendency toward higher levels of leisure-time physical activity at higher BMI was obvious. As shown in Table 3, after adjusting for additional confounders (Model 2, additionally adjusted for the other domains of physical activity), the patterns of the association between BMI and LTPA among strata of age group were similar.

**Fig 2:**
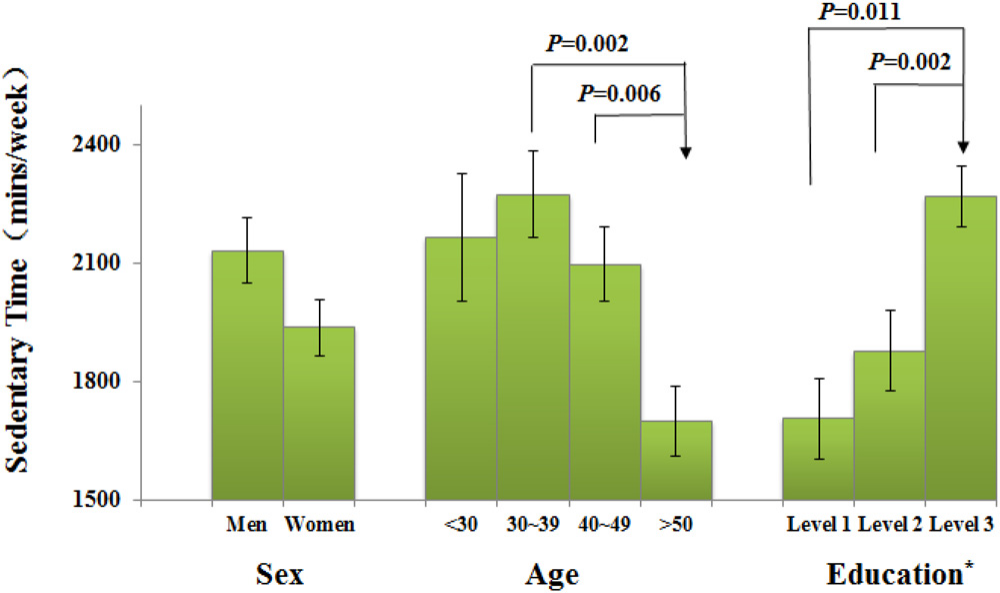
Differences in sedentary time associated with sex, age, and education level. The top line of each closed rectangle represents the adjusted mean, and the vertical bar represents its 95% CI, which was estimated using a multilevel mixed-effects model, after grouping by community. Education*: Level 1, Junior high school or below; Level 2, Senior high school; Level 3, College/university or above.

Fig 2 presents the multilevel regression results of sedentary behavior. There was no statistically significant effect of BMI on sedentary behavior (*P* = 0.450). Sedentary behavior was associated with sex (*P* = 0.001), age (*P* = 0.004) and attained educated level (*P*<0.001). Young men with the highest education level were found to sit more.

## Discussion

This study examined the varied associations of BMI with physical activity and sedentary behavior across socio-demographic groups in Chinese urban adults. Statistically significant associations between BMI and TPA/LTPA were not observed in this population as a whole, but these associations differed across sex, age and education level groups. Furthermore, there was no statistically significant association between BMI and sedentary time. To our knowledge, this is the first study focus on the modification of socio-demographic variables on the association between physical activity and BMI in China. This study also investigated the prevalence of sedentary behavior by gender, age, and education attainment level, as studies of sedentary behavior using regional or national representative data from China were limited.

As reported in recent studies, adults who were young and had a higher education level were more physically active than others in developed countries [22,23]. However, these findings are not consistent with what was observed in our study. In our survey, adults aged over 50 years and who had the least level of educational attainment were the most active and least sedentary. Our results are consistent with many of the studies conducted in China [4,17]. Educated young adults were more likely to be engaged in a sedentary job and work long hours facing high pressure. Conversely, people older than 50 years had more free time because of retirement or non-employment, and they paid more attention to their health status [24]. Furthermore, this study provided the physical activity levels within each of the four domains. There were no significant differences in the LTPA levels across the sex, age, and education level groups. The different distributions of TPA levels across the groups were primarily driven by work, transportation, and housework activities. Men aged 20–49 years had a significantly higher level of work-related physical activities than did others, whereas women aged 50–59 years with less education showed a higher level of transportation and housework activities.

In our study, the cross-sectional nature of the study limited our ability to make a causal inference for the association between BMI and PA. However, this may be an example of “reverse causation”, i.e., adiposity influences physical activity. We observed a positive association between BMI and physical activity among women and individuals older than 50 years. These uncommon positive associations are supported by the results from some studies, primarily in Western populations, that indicate two potential explanations: appearance motivation and health consciousness [11,13,24]. Moreover, it is elucidated clearly that these two potential motivations are varied across socio-demographic subgroups [11,12,14,15]. Women are significantly more dissatisfied with their BMI than are men. As people aged, the importance of appearance decreased, but consciousness of their health increased [24,25]. Limited evidence from China has consistently shown that appearance and health consciousness are the two primary motivations of overweight/obese people to participate in more physical activities [26,27]. It can explain our observations of the positive association between BMI and TPA among women. For these specific individuals, concern for body shape was more prevalent. Among individuals older than 50 years, the importance of appearance decreased, but consciousness of their health increased. Some of them, especially women, were retired or did not work. Therefore, for individuals aged older than 50 years with higher BMI who are eager to lose weight and keep healthy, LTPA is likely to be the first choice.

In our study, BMI were strongly and inversely associated with physical activity only in the 40–49 age groups. Several prospective studies indicated that overweight and obesity may lead to physical inactivity due to the discomfort experienced during physical exertion [6,7,8,28]. Furthermore, there has increased pressure on the people aged 40–49 years, particularly in urban. Meanwhile, they also be occupied with busy work and engaged in more social activities. It is difficult to make the exercise choice during their limited leisure time, especially heavier individuals. For them, physical activities may be something that take time, energy, and even lead to more stress.

Our results showed no significant association between BMI and sedentary time. Young men with highest educational level reported longer sitting times. In our study, we calculated a summary sitting variable, including the time spent sitting for work, transportation, and leisure. Sitting time during work, which was not totally controlled by personal willingness, most likely accounted for a large proportion. In addition, the current epidemiologic evidence has emphasized that the behavior of prolonged sitting was independent of physical activity [29,30]. Although some overweight and obese people participate in more physical activities, being motivated by appearance, health, or other reasons, they may still report prolonged sitting behavior. Thus far, the awareness of the health benefits of physical activity is much more prevalent and known among populations, while the benefits of reducing sitting, which are independent of increasing physical activity, are still less recognized.

While this study followed rigorous sampling strategy and quality control, several limitations must be considered. One of the limitations of this study was its cross-sectional study design. We cannot establish a causal association between TPA/LTPA and BMI. Second, physical activity and sedentary time were self-reported. Although the IPAQ-L has been shown to be valid for Chinese populations [31,32], information bias still exists because of subjective reporting. Some studies have reported that high BMI individuals tend to over-report their physical activity levels [4,33,34]. However, there was no evidence indicating that the over-report tendency differed by gender, age, or education level. Therefore, the observed positive associations between PA and BMI across some subgroups were unlikely be explained by reporting bias. On the other hand, if overweight and obesity individuals over-report their PA levels, it would weaken the inverse associations. Third, BMI was calculated from self-reported height and weight. However, most results of methodological research indicated that self-reported height, weight and BMI were generally valid and the differences between self-reported and measured data were acceptable in Asian adults [35,36,37]. Furthermore, height and weight are routine measurements during physical examinations in China. Finally, the study participants all came from three urban districts of Hangzhou, which have limited generalizability to all Chinese. In spite of these limitations, our results should have some implications for future studies and health policy makers for China.

## Conclusions

In general, socio-demographic characteristics modified the associations between physical activity and BMI among urban adults in Hangzhou. Furthermore, urban adults who were young and middle-aged and had high levels of education were most likely to live an inactive life. Pertinent physical activity promotion and sitting time reduction interventions are needed for individuals with different socio-demographic characteristics and varied exercise motivations.

## Supporting Information

S1 Dataset Data of this manuscript. (XLS)
